# Multispecies reservoir of *Spirometra erinaceieuropaei* (Cestoda: Diphyllobothridae) in carnivore communities in north-eastern Poland

**DOI:** 10.1186/s13071-020-04431-5

**Published:** 2020-11-10

**Authors:** Eliza Kondzior, Rafał Kowalczyk, Małgorzata Tokarska, Tomasz Borowik, Andrzej Zalewski, Marta Kołodziej-Sobocińska

**Affiliations:** 1 Mammal Research Institute, Polish Academy of Sciences, Stoczek 1, 17–230 Białowieża, Poland; 2 Faculty of Biology, University of Białystok, Ciołkowskiego 1J, 15–245 Białystok, Poland

**Keywords:** Sparganosis, Plerocercoid larvae, Paratenic hosts, European badger, Raccoon dog, Zoonosis

## Abstract

**Background:**

*Spirometra erinaceieuropaei* is a diphylobothriid tapeworm with a complex life-cycle including definitive, intermediate and paratenic (transport) hosts. Multiple routes of parasite transmission often make it impossible to determine what type of host a specific infected animal is considered to be. Spargana larvae cause sparganosis, a severe food- and water-borne disease mainly found in Asia. In Poland, *Spirometra* sp. was reported in large carnivores in Białowieża Primeval Forest for the first time in the 1940s and was recently confirmed as *S. erinaceieuropaei* in several mammals and snakes using molecular methods.

**Methods:**

In total, 583 carcasses of 9 carnivore species were necropsied between 2013 and 2019 in north-eastern (NE) Poland. The larvae of *S. erinaceieuropaei* (spargana) were isolated from subcutaneous tissue, counted, and preserved for genetic analyses. We calculated the prevalence and intensity of infection. To assess spatial variation in *S. erinaceieuropaei* infection probability in NE Poland, we applied a generalized additive model (GAM) with binomial error distribution. To confirm the species affiliation of isolated larvae, we amplified a partial fragment of the *18S* rRNA gene (240 bp in length).

**Results:**

*Spirometra* larvae were found in the subcutaneous tissue of 172 animals of 7 species and confirmed genetically as *S. erinaceieuropaei*. The overall prevalence in all studied hosts was 29.5% with a mean infection intensity of 14.1 ± 33.8 larvae per individual. Native European badgers and invasive raccoon dogs were characterized by the highest prevalence. An analysis of parasite spread showed a spatially diversified probability of infection with the highest values occurring in the biodiversity hot spot, Białowieża Primeval Forest.

**Conclusions:**

Our study revealed that various mammal species (both native and non-native) can serve as *S. erinaceieuropaei* reservoirs. The frequency and level of infection may differ between selected hosts and likely depend on host diversity and habitat structure in a given area. Further studies are needed to assess the distribution of the parasite throughout Europe and the environmental and biological factors influencing infection severity in wild mammals.
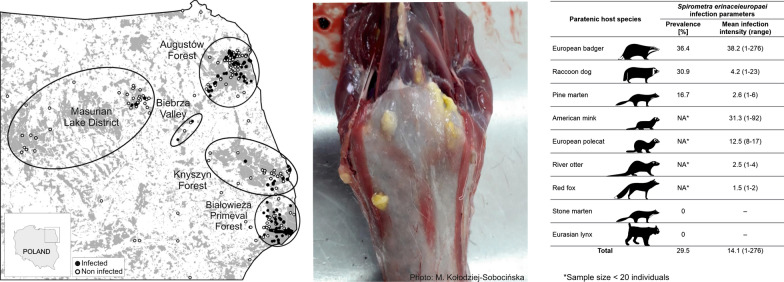

## Background

It is supposed that all organisms on Earth are involved in host-parasite interactions [1]. The trophic chain of hosts plays an important role for parasites in enabling them to come into contact with a variety of hosts and colonize new environments [2]. Parasite transmission is common among members of foraging guilds [3]. For most of helminth parasites, host specificity appears less defined at the intermediate host stage than at the definitive host stage, with non-adult intermediate (larval) stages able to infect different organs and tissues of diverse intermediate hosts. The range of specificity of the parasite is a crucial determinant of its invasive capacity and the likelihood of new host-parasite combinations occurring [4]. In addition, several parasites use paratenic (transport) hosts, where parasite larvae show no development [5]. The role of paratenic hosts is important; they facilitate contact between parasite larvae and the definitive host, contributing to an increase of prevalence in a specific host population [6].

*Spirometra* Faust, Campbell & Kellogg, 1929 is a genus of diphylobothriid cestodes that reproduce mainly in the small intestines of felids and canids [7, 8] and require two intermediate hosts. The unembryonated eggs released with animal faeces produce a ciliated stage (coracidium). The first intermediate host is a copepod (planktonic freshwater crustacean), in which coracidia develop into procercoid larvae (the first larval stage). When the infected copepod is swallowed by a second intermediate host, such as an amphibian or reptile [9–12], the procercoid larvae penetrate the intestinal tract and transform to plerocercoids (spargana), which then migrate and settle in other organs and tissues, such as subcutaneous connective tissue, the brain, lungs, spinal cord, urinary bladder or eye [13–20].

The life-cycle of *Spirometra* spp. may also include paratenic hosts. Second intermediate hosts infected with plerocercoid larvae can be preyed upon and thus reach a wide variety of tetrapods such as birds or mammals (including humans) that may serve as paratenic hosts for this parasite [21, 22]. Multiple routes of parasite transmission often make it impossible to determine what type of host a specific infected animal is considered to be. After passing through the intestinal wall, spargana settle in host tissues and cause sparganosis [23, 24]. Paratenic hosts, however, are unnecessary for the completion of life-cycle, thus the larval *Spirometra* can infect and subsist in numerous species of paratenic hosts until finally consumed by felids or canids, serving as definitive hosts [25], though they are also reservoirs of spargana for other carnivores. It has also been found that some canids, such as the red fox (*Vulpes vulpes* Linnaeus, 1758) and the raccoon dog (*Nyctereutes procyonoides* Gray, 1834), may serve as both definitive and paratenic hosts [7, 26, 27].

Spargana in intermediate and/or paratenic hosts cause sparganosis, a severe food- and water-borne disease [18, 23, 28]. Most of the research on sparganosis has been conducted in Asia, where sparganosis is a serious public health problem [11, 13–17, 19, 22, 23]. However, there are also reports from other continents, including South and North America, Africa, Australia, and Europe [29–31]. European records of sparganosis in wildlife are mainly based on incidental reports of the presence of the parasite in vertebrates, including canids [26, 27], mustelids [32–34], rodents [35], insectivores [7], snakes [9, 36, 37] and frogs [36–38]. In Poland, the first report of adult *Spirometra* was described as *S. janickii* in wolves (*Canis lupus* Linnaeus, 1758) and Eurasian lynxes (*Lynx lynx* Linnaeus, 1758) from Białowieża Primeval Forest (BPF) in the 1940s [7]. Nonetheless, this finding is controversial. This species has not been reported since its original description, likely because most authors synonymised *S. janickii* with *S. erinaceieuropaei*. European genotypes of *S. erinaceieuropaei* in Polish wildlife were reported for several species of mammals, including the American mink (*Neovison vison* Schreber, 1777) [39], Eurasian lynx [8], European badger (*Meles meles* Linnaeus, 1758*)* [32], European polecat (*Mustela putorius* Linnaeus, 1758) [39], raccoon dog [39] and wild boar (*Sus scrofa* Linnaeus, 1758) [40], and for one species of reptile, the grass snake (*Natrix natrix* Linnaeus, 1758) [9]. Recently, the first case of human sparganosis in Poland was confirmed in an individual in the surroundings of BPF [28]; the source of infection likely being consumption of wild boar meat containing spargana [40]. It can be supposed that the consumption of venison may cause an increase in the number of cases of human sparganosis.

The role of wild carnivores as transmission vectors for zoonotic diseases has been widely described [41–44] and includes both native as well as non-native invasive species. Invasive species are important for spreading and transmitting of diseases because they can carry their own parasites and acquire new ones during the colonization of new territories [45, 46]. Nothing is known about the role of invasive species in the spread of sparganosis, and very little about the contribution of native carnivores to the maintenance of this disease in European wildlife.

The main goal of this paper was to investigate the spread of *S. erinaceieuropaei* in well-preserved communities of wild carnivores in north-eastern (NE) Poland. We aimed to: (i) investigate which carnivore species (native and/or non-native invasive) may serve as *S. erinaceieuropaei* hosts in wildlife and whether the parasite’s presence is only local or perhaps more widespread; (ii) calculate the infection parameters, prevalence and intensity; (iii) assess spatial patterns of infection probability in the study area; and (iv) reveal whether wild canids and felids, the typical definitive hosts, may also serve as paratenic hosts carrying spargana.

## Methods

### Study area

Our study was performed in Podlasie and the eastern part of the Masurian Lakes region, north-eastern (NE) Poland. Both regions are characterized by well-preserved forest ecosystems (with four national parks and a large area covered by Natura 2000, a network of nature protection areas in the territory of the European Union) and greater forest cover (32%) in comparison to the rest of Poland [47]. Our main sampling sites were large forested areas: Augustów Forest (AF); Białowieża Primeval Forest (BPF); Biebrza Valley (BV); Knyszyn Forest (KF); and the Masurian Lake District (MLD). AF, KF and MLD are dominated by coniferous forests [48]. In BPF over 60% of the area is covered by deciduous and mixed forests with high species richness [49]. Wet habitats cover over 40% of the area [48], and BV is the largest marshland in central Europe with concentrated patches of woodlands [50]. Moreover, the northern part of the study area in MLD and AF is interspersed by numerous lakes.

The region is characterized by a low degree of urbanization and the lowest human densities in Poland (59 individuals/km^2^) [47]. NE Poland is located within a zone of temperate transitional climate with marked continental influences. The study area is inhabited by well-preserved animal communities with as many as 12 local species of carnivores [51].

### Carcass collection

**Fig. 1:**
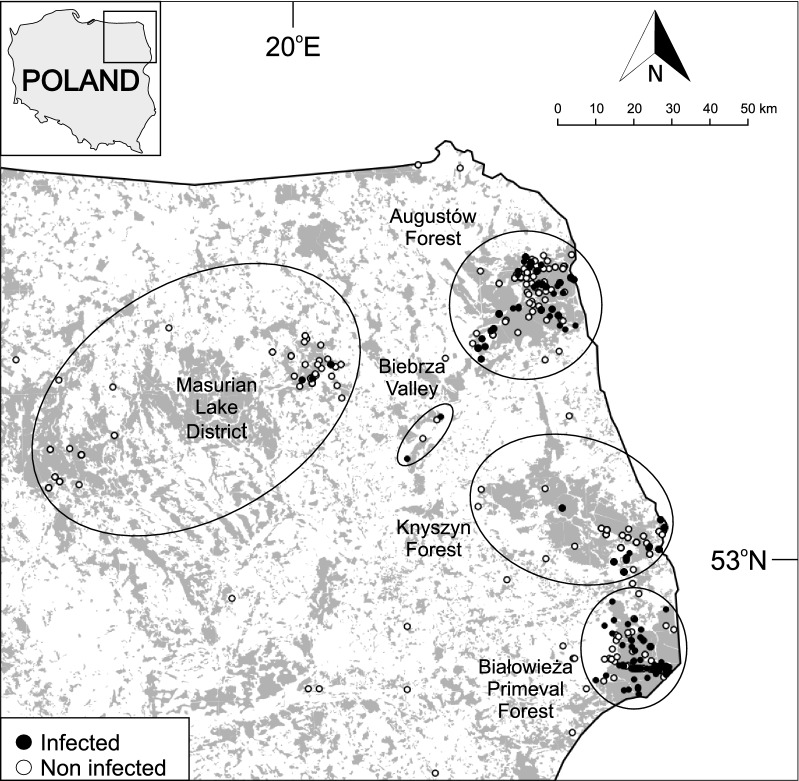
The distribution of study sites and samples in the study area. Black dots indicate carnivore individuals infected with *Spirometra erinaceieuropaei*, white dots indicate non-infected individuals

A total of 583 carcasses of 9 mammal species were collected in BPF and AF between 2013 and 2019, in BV, KF and MLD between 2016 and 2019, and in the surroundings of studied forests between 2015 and 2019 (Fig. 1, Table 1). Mammal carcasses originated from road kills or legal culling from predator control for gallinaceous bird, i.e. the black grouse (*Lyrurus tetrix* Linnaeus, 1758) and the western cappercaillie (*Tetrao urogallus* Linnaeus, 1758), conservation projects. No animals were killed specifically for this study.

**Table 1:** Prevalence and infection intensity of *Spirometra erinaceieuropaei* in carnivores in NE Poland

Species	Location of hosts (no. of infected individuals in parentheses)						Sample size	No. of infected	Prevalence (%)	95% CI	Mean infection intensity (range)
	AF	BPF	BV	KF	MLD	Other					
Raccoon dog (*Nyctereutes procyonoides*)*	123 (37)	90 (59)	4 (1)	71 (9)	64 (3)	1 (0)	353	109	30.9	26.1–35.8	4.2 (1–23)
European badger (*Meles meles*)	87 (31)	19 (13)	–	5 (3)	14 (0)	4 (0)	129	47	36.4	28.4–44.8	38.2 (1–276)
Pine marten (*Martes martes*)	11 (0)	22 (7)	–	4 (0)	–	5 (0)	42	7	16.7	7.0–28.8	2.6 (1–6)
Stone marten (*Martes foina*)	5 (0)	11 (0)	–	6 (0)	3 (0)	2 (0)	27	0	0	0–0.07	–
Red fox (*Vulpes vulpes*)	–	7 (2)	–	7 (0)	1 (0)	–	15	2	na	na	1.5 (1–2)
European polecat (*Mustela putorius*)	1 (0)	1 (1)	–	1 (1)	–	3 (0)	6	2	na	na	12.5 (8–17)
American mink (*Neovison vison*)*	1 (0)	3 (3)	–	–	–	–	4	3	na	na	31.3 (1–92)
River otter (*Lutra lutra*)	–	3 (1)	1 (0)	–	–	–	4	2	na	na	2.5 (1–4)
Eurasian lynx (*Lynx lynx*)	1 (0)	1 (0)	–	1 (0)	–	–	3	0	na	na	–
Total	229 (68)	157 (86)	5 (1)	95 (13)	82 (3)	15 (0)	583	172	29.5	25.9–33.2	14.1 (1–276)

*Notes*: Prevalence was calculated only for species with sample size > 20 individuals. Locations: AF, Augustów Forest; BPF, Białowieża Primeval Forest; BV, Biebrza Valley; KF, Knyszyn Forest; MLD, Masurian Lake District; Other, areas surrounding studied forest. Binomial confidence intervals were calculated using Bayesian inference

Invasive species are marked with an asterisk

*Abbreviations*: CI, confidence interval; na, not available

### Necropsies and genetic identification of larvae

The collected carcasses were kept frozen at − 80 °C for a minimum of one week to minimize the risk of *Echinococcus multilocularis* (Leuckart, 1863) infection [52]. During necropsies the animals were weighed, measured and sexed. Spargana were isolated from subcutaneous tissue, counted, and preserved in 99% ethanol for molecular identification as described below. Prevalence (the proportion of infected individuals in %) was calculated as the ratio of infected individuals to all collected study animals. The mean intensity of infection in each host species was calculated as the average number of spargana per one infected individual of a particular species. We also calculated a standard deviation of the mean intensity of infection as the square root of its variance.

To confirm larval species affiliation, we molecularly confirmed 25 larvae isolated from different carnivore species (see details in Additional file 1: Table S1, Additional file 2: Figure S1, Additional file 3: Figure S2). We used primers and procedures described by Liu et al. [53] to achieve a sequence of an evolutionarily conserved nuclear *18S* rRNA gene of over 240 bp in length. The sequences were aligned with previously analysed ones from wild boars, European badgers, and grass snakes from BPF [9, 32, 40] (Additional file 1: Table S1), as well as with sequences available on GenBank subsequently trimmed to 222 bp in length and analysed using the ClustalW multiple alignment test (BioEdit sequence alignment editor [54]) and Basic Local Alignment Search Tool (BLAST) [55]. A Tamura-Neil model (TrN) was selected for maximum likelihood analysis using MEGA v6 [56]. The following reference sequences for other Diphyllobothriidae were retrieved from GenBank: *Spirometra erinacei* (D64072.1); *S. erinaceieuropaei* (KX528090 and KX552801); and *Dibothriocephalus latus* (Linnaeus, 1758) (KF218247, KF218246.1 and DQ925309). *Taenia pisiformis* (Bloch, 1780) (JX317675.1) and *Taenia krabbei* (Moniez, 1879) (MH843684.1) were used as the outgroup in analyses (Additional file 2: Figure S1 and Additional file 3: Figure S2). A list of all newly generated sequences of *18S* rRNA is presented in Additional file 1: Table S1.

### Statistical analysis

To assess the spatial variation of *S. erinaceieuropaei* infection probability in NE Poland, we applied a generalized additive model (GAM) with binomial error distribution in the *mgcv* package implemented in R [57]. We added the presence/absence of larvae in the sampled individuals as a binomial dependent variable, while the interaction of longitude and latitude of sample locations (explanatory variables) was fitted as a non-parametric spline. We limited analyses to samples collected in BPF, KF, AF and BV due to larger numbers of carcasses collected during the study period.

## Results

**Fig. 2:**
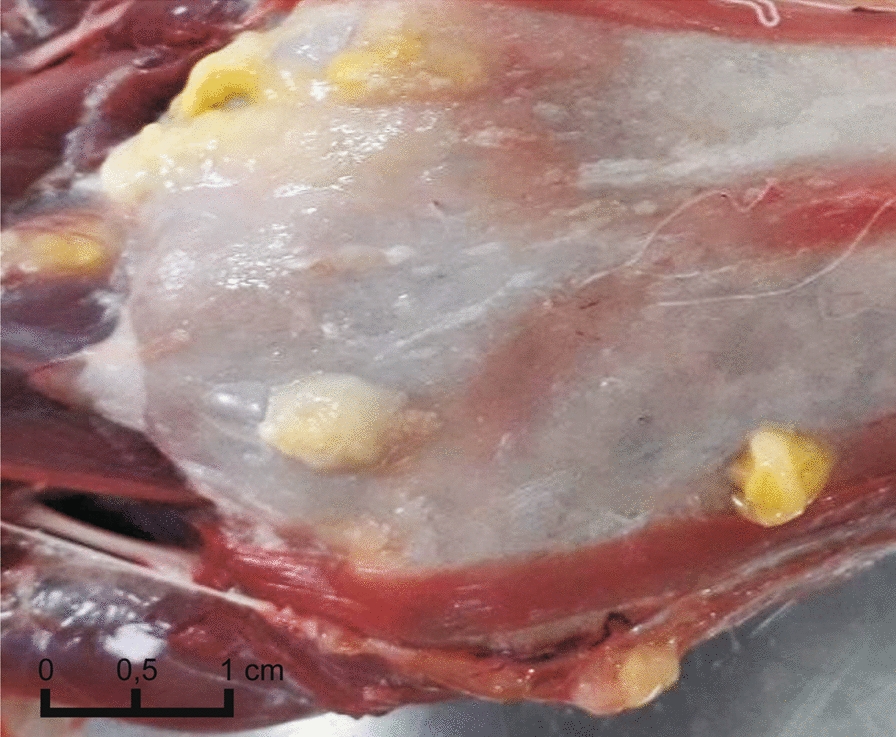
Cysts containing *Spirometra erinaceieuropaei* plerocercoids in subcutaneous tissue of American mink (*Neovison vison*) from Białowieża Primeval Forest. Visible skin from the inside in the abdominal part of the body

Plerocercoid larvae of *Spirometra* were found in 172 of 583 mammals belonging to 7 out of 9 studied species (Table 1, Fig. 2). The overall prevalence of spargana infection in carnivore hosts was 29.5%. The highest overall prevalence was estimated for European badgers (36.4%) and raccoon dogs (30.9%). Twenty-seven stone martens (*Martes foina* Erxleben, 1777) and 3 Eurasian lynxes were uninfected. The overall mean infection intensity was 14.1 ± 33.8 with the highest values up to 276 found in European badgers (38.2 ± 56.0) (Table 1).

All 25 newly generated *18S* rRNA sequences of larvae isolated from autopsied animals of 7 different species showed 99% identity with the 3 sequences for *S. erinaceieuropaei* deposited on GenBank (Additional file 2: Figure S1, Additional file 3: Figure S2). Comparisons with previously published *18S* rRNA sequences from isolates in European badgers, wild boars, and grass snakes, revealed that the new sequences all showed 99.4–100% similarity (BLAST). The only variation was observed in the DNA of 2 out of 4 analysed larvae originating from European polecats and was, in both cases, an insertion of a T nucleotide at position 207 of sequences (see Additional file 2: Figure S1). The occurrence of *S. erinaceieuropaei* was confirmed for the first time using molecular methods in the pine marten (*Martes martes* Linnaeus, 1758), red fox, and river otter (*Lutra lutra* Linnaeus, 1758) (Additional file 1: Table S1; Additional file 2: Figure S1, Additional file 3: Figure S2).

**Fig. 3:**
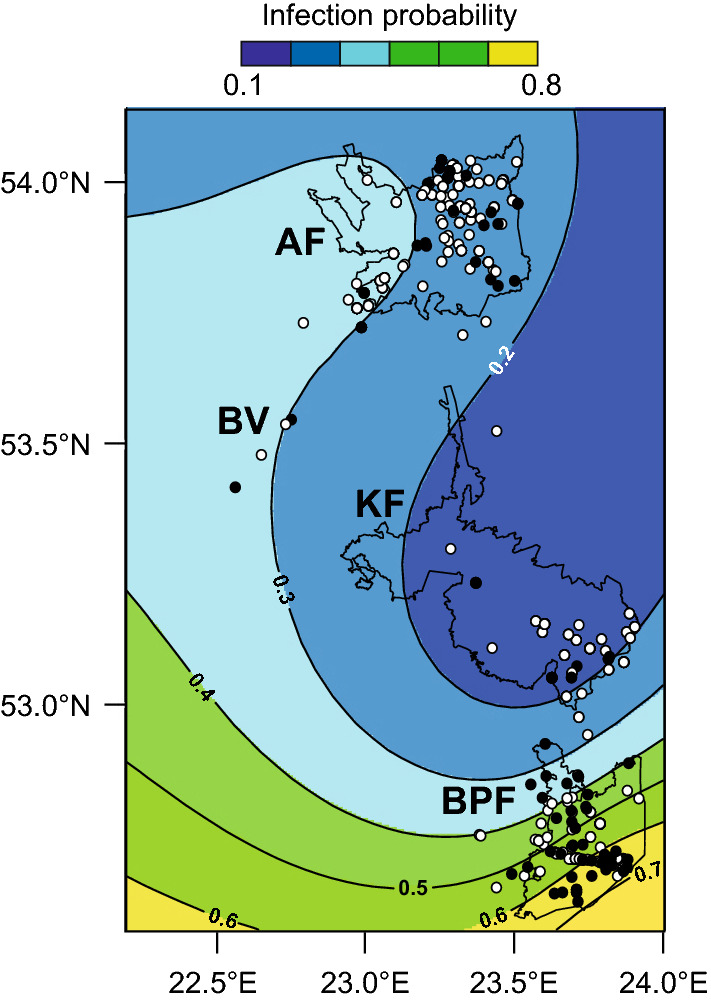
The predicted probability of *Spirometra erinaceieuropaei* infection in carnivore hosts in NE Poland. Results of the generalized additive model (GAM). Black dots indicate infected animals, white dots indicate non-infected animals. *Abbreviations*: AF, Augustów Forest; BPF, Białowieża Primeval Forest; KF, Knyszyn Forest; BV, Biebrza Valley

The GAM showed a significant spatial pattern in the probability of *S. erinaceieuropaei* infection in the study area (*χ*^2^ = 47.8, *P* < 0.001). The highest probability of infection was observed in BPF and was observed to decrease northward (Fig. 3).

## Discussion

Most European cases of sparganosis in wildlife represent exclusively single findings. Thus far, the only comprehensive research has been carried out in Belarusian Polesie, which is closely related to BPF where the parasite has been found in amphibians, reptiles and mammals (including 7 species of carnivores) [26, 27, 33, 37, 58–61]. Our study focused on *S. erinaceieuropaei* spread in well-preserved carnivore communities of NE Poland. We revealed high species richness of intermediate/paratenic mammalian hosts for *S. erinaceieuropaei*, including seven out of nine studied species, both native (European badger, European polecat, river otter, pine marten and red fox) and invasive (American mink and raccoon dog). Previously, the parasite was also confirmed in Poland in the wild boar (larvae) as well as in the Eurasian lynx and wolf (adult parasites) [7, 8, 32, 39, 40]. We molecularly confirmed pine marten and river otter as hosts of *S. erinaceieuropaei* for the first time and provided the first comprehensive epidemiological data for seven mammal species in NE Poland. Previous research from Europe has also shown six other mammal species as hosts of *S. erinaceieuropaei*, including the brown rat (*Mus decumanus* Berkenhout, 1769), European hedgehog (*Erinaceus europeaus* Linnaeus, 1758), European mink (*Mustela lutreola* Linnaeus, 1761), European mole (*Talpa europaea* Linnaeus, 1758), stoat (*Mustela erminea* Linnaeus, 1758) and weasel (*Mustela nivalis* Linnaeus, 1766) [34, 35, 60–62]. The species richness of infected carnivores indicates a wider than previously expected spread and complex circulation of the parasite in wildlife. The lack of other comprehensive data about *S. erinaceieuropaei* occurrence in Europe has previously not given us a reason to suppose that the parasite is so widespread and prevalent in European wildlife.

The highest infection rates were revealed in the invasive raccoon dog and the native European badger. Therefore, the role of invasive species may be particularly significant in disease spreading; by settling into new areas, they can either bring alien parasite species into colonized areas [44, 63], or become new hosts for native parasites and facilitate their spread [44]. High infection rates in both species may result from consuming prey (primarily amphibians and reptiles) in addition to carrion (wild boar and other mesocarnivores) for the raccoon dog that serve as the source of *Spirometra* infection [51]. Moreover, European badgers and raccoon dogs are preyed upon and consumed by large predators (wolf and Eurasian lynx) [51, 64]; predation by wolves and dogs is one of the main sources of raccoon dog mortality [64]. This allows *Spirometra* to complete its life-cycle and continue spreading. The parasite can be effectively spread over large areas by its medium-sized and large carnivores hosts (European badger: maximal daily movement distance 17.5 km; Eurasian lynx: 24.8 km; wolf: 64 km) [65–67], or even to human settlements through dogs. Further studies are needed to reveal how diet composition influences infection severity in *Spirometra*-infected hosts.

We did not find *S. erinaceieuropaei* plerocercoids in stone marten and Eurasian lynx specimens. This could be due to stone marten habitat selection as well as the diet of both species. The stone martens’ diet does not consist of amphibians and reptiles; they mainly prey on small mammals and birds [68], which can be a potential source of infection. In NE Europe, stone martens usually occur in urban and rural areas and avoid large, continuous forest complexes [69], which limit their potential to come into contact with possible wild hosts of *S. erinaceieuropaei*. So far, stone martens have been considered a host for *Spirometra* sp. accidentally in Italy [70]. However, the Eurasian lynx is a known definitive host of *S. erinaceieuropaei* [7, 8, 71]. Its diet only sporadically consists of amphibians, and it mostly preys on ungulates, which do not serve as hosts for *Spirometra* [51]. Moreover, carnivores like the red fox, raccoon dog and raccoon (*Procyon lotor* Linnaeus, 1758) may serve as both intermediate/paratenic and definitive host of *Spirometra* sp. [26, 27, 72]. We did not find any spargana in the three Eurasian lynx carcasses, but the number of studied animals was very low. Raccoons have not yet been reported as either intermediate/paratenic or definitive host for *Spirometra* in European wildlife; they have only been confirmed experimentally [72]. However, our study has confirmed that the red fox and raccoon dog, the typical definitive hosts, can also act as intermediate/paratenic hosts for *S. erinaceieuropaei*. This may be important for sparganosis spread in wildlife, since it highlights the diversity of the disease transmission routes, which range from parasite eggs present in the faeces of definitive hosts, to intermediate/paratenic hosts infected with spargana and preyed upon by large predators [46, 64].

We focused on intermediate/paratenic hosts for *S. erinaceieuropaei* in which larvae may be found in subcutaneous tissue and other organs. Morphological identification of spargana to the species level is impossible and such analyses are justified only in the case of adult tapeworms that complete their life-cycle in a definitive host [24]. Thus, it is highly recommended to use molecular methods for reliable identification of larval *Spirometra*. So far, notwithstanding our studies in NE Poland over the last few years [8, 9, 32, 39, 40], no genetic analyses of *Spirometra* larvae in Europe have been carried out; therefore, it is not clear which species have caused previously reported infections. Additionally, the adult tapeworms of *S. janickii* described by Furmaga in the 1950s [7], have not yet been found again, and thus cannot be analysed in more detail. Our study revealed only minor genetic divergence in the studied *18S* rRNA gene sequences, which can be interpreted as inter-individual differences. All studied parasite specimens isolated from different hosts and locations undoubtedly belong to the same species, *S. erinaceieuropaei*. This may be explained by the fact that the sites are separated by no more than 200 km and hosts such as the Eurasian lynx, wolf, red fox, raccoon dog, and European badger can move over significant distances, dispersing parasites between sites [73–76]. Our study showed that the probability of *S. erinaceieuropaei* infection varies spatially. The highest probability of infection and prevalence (54.8%) occurred in BPF, indicating beneficial conditions for the parasite, likely because this site had the highest species richness of mammal species among all studied sites [49, 51, 77]. Additionally, *S. erinaceieuropaei* was found in grass snakes from BPF [9], which may promote the transmission of sparganosis in wildlife in this area. It was found that a decrease in the number of intermediate/paratenic hosts results in a lower prevalence in definitive hosts [78]; thus, host diversity may be an important factor responsible for the spread of sparganosis. Mammalian species richness was lower in other locations [48, 51], although we observed that the probability of infection was highest inside forest complexes and decreased as the distance from the forest complex increased. BPF was also characterized by various forest and non-forest habitats, including wetlands [48], which are crucial to the life-cycle of *Spirometra* [10, 24]. Wet habitats in BPF are the optimal environment for the development of freshwater copepods, which are the first intermediate hosts for the parasite. In *S. erinaceieuropaei* hosts inhabiting AF and BV, the probability of infection was lower than in BPF, but higher than in KF. This may be due to lower habitat heterogeneity, but with a relatively high percentage of wetland habitats [48]. KF, characterized by the lowest probability of infection and prevalence (13.7%), is generally less diversified in terms of species and habitat structure, with a lower proportion of wetlands [48, 49].

## Conclusions

Our study revealed that the spectrum of *S. erinaceieuropaei* intermediate/paratenic hosts in Europe is broad, and therefore, that the trophic dependencies that enable *S. erinaceieuropaei* to spread in the environment are complex. Sparganosis is likely more widespread in European wildlife than expected and may be transmitted by both native and not-native mammals. Presumably, infection rates vary spatially and depend on numerous factors, including habitat structure, species richness, and density of potential hosts. Further research is required to confirm which environmental and biological factors have the most significant impact on shaping the level of *S. erinaceieuropaei* infection in European wild mammalian hosts.

## Supplementary information

**Additional file 1: Table S1.** *Spirometra erinaceieuropaei* larvae isolated from different mammalian host species which were analysed genetically with accession numbers of obtained sequences. *Abbreviations*: BPF, Białowieża Primeval Forest; KF, Knyszyn Forest.

**Additional file 2: Figure S1.** *18S* rRNA gene fragment (222 bp) alignment of *Spirometra erinacei* (GenBank: D64072.1, KX528090 and KY552801) and DNA of *Spirometra* individuals extracted from 9 different mammal species combined with 3 related plathyhelminth species, *Diphyllobothrium latum*, *Taenia krabbei* and *Taenia pisiformis.* The newly generated sequences are indicated in bold; the sequences of *Spirometra* from the European badger, wild boar, and grass snake have been published in 2014, 2016 and 2018 [9, 32, 40]. Dots indicate nucleotide identity with the reference sequence (Polecat 50L_4). The alignment shows almost complete genetic homogeneity in most cases of *Spirometra erinaceieuropaei* from various mammalian species from north-eastern Poland.

**Additional file 3: Figure S2.** Maximum likelihood phylogenetic tree of 225 bp sequences of the *18S* RNA gene fragment based on 38 sequences of *Spirometra* sp. individuals extracted from mammal and reptile species and reference sequences retrieved from GenBank: *Spirometra erinacei* (D64072.1); *Taenia krabbei* (MH843684.1); and *Taenia pisiformis* (JX317675.1). The sequences generated in the present study are indicated in bold. *Spirometra* DNA sequences from the European badger, wild boar, and grass snake had been published by Kołodziej-Sobocińska et al. [32, 40] and Kondzior et al. [9]. The tree is drawn to scale, the scale-bar indicates the number of substitutions per site.

## Data Availability

The sequences analysed during the presented study are deposited in the GenBank database under the accession numbers MT127121-MT127125, MT131358-MT131361, MT136495-MT136508, MT140351 and MT140352.

## Abbreviations

AF: Augustów Forest
BLAST: Basic Local Alignment Search Tool
BV: Biebrza Valley
BPF: Białowieża Primeval Forest
GAM: generalized additive model
KF: Knyszyn Forest
MLD: Masurian Lake District
NE: North-eastern
