# Supplementary material for: Multispecies reservoir of Spirometra erinaceieuropaei (Cestoda: Diphyllobothridae) in carnivore communities in north-eastern Poland
Source: Parasit Vectors. 2020 Nov 10;13:560. doi: 10.1186/s13071-020-04431-5 (PMC7654582; doi:10.1186/s13071-020-04431-5)
Supplement: Supplementary file 1 — Additional file 1: Table S1. Spirometra erinaceieuropaei larvae isolated from different mammalian host species which were analysed genetically with accession numbers of obtained sequences. Abbreviations: BPF, Białowieża Primeval Forest; KF, Knyszyn Forest. [file 13071_2020_4431_MOESM1_ESM.docx]

# Multispecies reservoir of *Spirometra erinaceieuropaei* (Cestoda: Diphyllobothridae) in carnivore communities in north-eastern Poland

Eliza Kondzior^1,2*^, Rafał Kowalczyk^1^, Małgorzata Tokarska^1^, Tomasz Borowik^1^, Andrzej Zalewski^1^, Marta Kołodziej-Sobocińska^1^

^1^ Mammal Research Institute, Polish Academy of Sciences, Stoczek 1, 17-230 Białowieża, Poland

^2^ Faculty of Biology, University of Białystok, Ciołkowskiego 1J, 15-245 Białystok, Poland

**Additional file 1:** Table S1. *Spirometra erinaceieuropaei* larvae isolated from different mammalian host species which were analysed genetically with accession numbers of obtained sequences. BPF – Białowieża Primeval Forest, KF – Knyszyn Forest.

| Host species | Sample ID | Locality Code | Accesion number  GenBank |
| --- | --- | --- | --- |
| European badger  *Meles meles* | 2B_1  2B_2  2B_3  2B_4  2B_5 | BPF  BPF  BPF  BPF  BPF | MT127121  MT127122  MT127123  MT127124  MT127125 |
| River otter  *Lutra lutra* | W1_1  W1_2 | BPF  BPF | MT140352  MT140351 |
| Raccoon dog  *Nyctereutes procyonoides* | J1_1  J1_2 | BPF  BPF | MT136502  MT136501 |
| Red fox  *Vulpes vulpes* | 1L  2L | BPF  BPF | MT136508  MT136507 |
| American mink  *Neovison vison* | 1N  73L_1b  73L_2  73L_4 | BPF  BPF  BPF  BPF | MT136503  MT136506  MT136505  MT136504 |
| Pine marten  *Martes martes* | 51L_1  51L_2  54L_1  54L_2  54L_3  54L_4 | BPF  BPF  BPF  BPF  BPF  BPF | MT136500  MT136497  MT136499  MT136498  MT136496  MT136495 |
| European polecat  *Mustela putorius* | 50L_1  50L_2  50L_3  50L_4 | KF  KF  KF  KF | MT131361  MT131360  MT131359  MT131358 |
