# Supplementary material for: Multispecies reservoir of Spirometra erinaceieuropaei (Cestoda: Diphyllobothridae) in carnivore communities in north-eastern Poland
Source: Parasit Vectors. 2020 Nov 10;13:560. doi: 10.1186/s13071-020-04431-5 (PMC7654582; doi:10.1186/s13071-020-04431-5)
Supplement: Supplementary file 2 — Additional file 2: Figure S1. 18S rRNA gene fragment (222 bp) alignment of Spirometra erinacei (GenBank: D64072.1, KX528090 and KY552801) and DNA of Spirometra individuals extracted from 9 different mammal species combined with 3 related plathyhelminth species, Diphyllobothrium latum, Taenia krabbei and Taenia pisiformis. The newly generated sequences are indicated in bold; the sequences of Spirometra from the European badger, wild boar, and grass snake have been published in 2014, 2016 and 2018 [9, 32, 40]. Dots indicate nucleotide identity with the reference sequence (Polecat 50L_4). The alignment shows almost complete genetic homogeneity in most cases of Spirometra erinaceieuropaei from various mammalian species from north-eastern Poland. [file 13071_2020_4431_MOESM2_ESM.docx]

# Multispecies reservoir of *Spirometra erinaceieuropaei* (Cestoda: Diphyllobothridae) in carnivore communities in north-eastern Poland

Eliza Kondzior^1,2*^, Rafał Kowalczyk^1^, Małgorzata Tokarska^1^, Tomasz Borowik^1^, Andrzej Zalewski^1^, Marta Kołodziej-Sobocińska^1^

^1^ Mammal Research Institute, Polish Academy of Sciences, Stoczek 1, 17-230 Białowieża, Poland

^2^ Faculty of Biology, University of Białystok, Ciołkowskiego 1J, 15-245 Białystok, Poland

**Additional file 2: Figure S1.** 18S rRNA gene fragment (222 bp) alignment of *Spirometra erinacei* (GenBank access ID: D64072.1, KX528090, KY552801) and DNA of *Spirometra*individuals extracted from 9 different mammal species combined with 3 related plathyhelminthes species: *Diphyllobothrium latum*, *Taenia krabbei* and *Taenia pisiformis.* The names of sequences acquired in this study are bold, European badger, wild boar, and grass snake-achieved Spirometra DNA sequences had been described in the 2014, 2016 and in 2018 [9,32,40]. Dotted sequences indicate nucleotide identity with the reference sequence (Polecat 50L_4). The alignment shows almost complete genetic homogeneity in most cases of *Spirometra erinaceieuropaei* from various mammalian species from north-eastern Poland.

10 20 30 40 50 60 70 80 90

....|....| ....|....| ....|....| ....|....| ....|....| ....|....| ....|....| ....|....| ....|....|

Badger *Meles meles*1B_1 **..........** **..........** **......----** **---.......** **......--..** **..........** **.......-..** **....-----.** **..........**

Badger *Meles meles*1B_2 **..........** **..........** **......----** **---.......** **......--..** **..........** **.......-..** **....-----.** **..........**

Badger *Meles meles*1B_3 **..........** **..........** **......----** **---.......** **......--..** **..........** **.......-..** **....-----.** **..........**

**Badger *Meles meles*2B_1** **..........** **..........** **......----** **---.......** **......--..** **..........** **.......-..** **....-----.** **..........**

**Badger *Meles meles*2B_2** **..........** **..........** **......----** **---.......** **......--..** **..........** **.......-..** **....-----.** **..........**

**Badger *Meles meles*2B_3** **..........** **..........** **......----** **---.......** **......--..** **..........** **.......-..** **....-----.** **..........**

**Badger *Meles meles*2B_4** **..........** **..........** **......----** **---.......** **......--..** **..........** **.......-..** **....-----.** **..........**

**Badger *Meles meles*2B_5** **..........** **..........** **......----** **---.......** **......--..** **..........** **.......-..** **....-----.** **..........**

Wild boar *Sus scrofa*1D **..........** **..........** **......----** **---.......** **......--..** **..........** **.......-..** **....-----.** **..........**

Wild boar *Sus scrofa*2D **..........** **..........** **......----** **---.......** **......--..** **..........** **.......-..** **....-----.** **..........**

Wild boar *Sus scrofa*3D **..........** **..........** **......----** **---.......** **......--..** **..........** **.......-..** **....-----.** **..........**

Grass snake *Natrix natrix*41G **..........** **..........** **......----** **---.......** **......--..** **..........** **.......-..** **....-----.** **..........**

Grass snake *Natrix natrix*53G_1 **..........** **..........** **......----** **---.......** **......--..** **..........** **.......-..** **....-----.** **..........**

Grass snake *Natrix natrix*53G_2 **..........** **..........** **......----** **---.......** **......--..** **..........** **.......-..** **....-----.** **..........**

Grass snake *Natrix natrix*53G_3 **..........** **..........** **......----** **---.......** **......--..** **..........** **.......-..** **....-----.** **..........**

**Otter *Lutra lutra*W1_1** **..........** **..........** **......----** **---.......** **......--..** **..........** **.......-..** **....-----.** **..........**

**Otter *Lutra lutra*W1_2** **..........** **..........** **......----** **---.......** **......--..** **..........** **.......-..** **....-----.** **..........**

**Raccoon dog *N.procyonoides*J1_1** **..........** **..........** **......----** **---.......** **......--..** **..........** **.......-..** **....-----.** **..........**

**Raccoon dog *N.procyonoides*J1_2** **..........** **..........** **......----** **---.......** **......--..** **..........** **.......-..** **....-----.** **..........**

**Fox *Vulpes vulpes*Lis 1** **..........** **..........** **......----** **---.......** **......--..** **..........** **.......-..** **....-----.** **..........**

**Fox *Vulpes vulpes*Lis_2** **..........** **..........** **......----** **---.......** **......--..** **..........** **.......-..** **....-----.** **..........**

**Am. mink *Neovison vison*1N_1** **..........** **..........** **......----** **---.......** **......--..** **..........** **.......-..** **....-----.** **..........**

**Am. mink *Neovison vison*73L_1b** **..........** **..........** **......----** **---.......** **......--..** **..........** **.......-..** **....-----.** **..........**

**Am. mink *Neovison vison*73L_2** **..........** **..........** **......----** **---.......** **......--..** **..........** **.......-..** **....-----.** **..........**

**Am. mink *Neovison vison*73L_4** **..........** **..........** **......----** **---.......** **......--..** **..........** **.......-..** **....-----.** **..........**

**Pine marten *Martes martes*51L_**1 .......... .......... ......---- ---....... ......--.. .......... .......-.. ....-----. ..........

**Pine marten *Martes martes*51L_2** **..........** **..........** **......----** **---.......** **......--..** **..........** **.......-..** **....-----.** **..........**

**Pine marten *Martes martes*54L_1** **..........** **..........** **......----** **---.......** **......--..** **..........** **.......-..** **....-----.** **..........**

**Pine marten *Martes martes*54L_2** **..........** **..........** **......----** **---.......** **......--..** **..........** **.......-..** **....-----.** **..........**

**Pine marten *Martes martes*54L_3** **..........** **..........** **......----** **---.......** **......--..** **..........** **.......-..** **....-----.** **..........**

**Pine marten *Martes martes*54L_4** **..........** **..........** **......----** **---.......** **......--..** **..........** **.......-..** **....-----.** **..........**

**Polecat *Mustela putorius*50L_1** **..........** **..........** **......----** **---.......** **......--..** **..........** **.......-..** **....-----.** **..........**

**Polecat *Mustela putorius*50L_2** **..........** **..........** **......----** **---.......** **......--..** **..........** **.......-..** **....-----.** **..........**

**Polecat *Mustela putorius*50L_3** **..........** **..........** **......----** **---.......** **......--..** **..........** **.......-..** **....-----.** **..........**

**Polecat *Mustela putorius*50L_4** **CTTGCGCTGA** **TTACGTCCCT** **GCCCTT----** **---TGTACAC** **ACCGCC--CG** **TCGCTACTAC** **CGATTGA-AT** **GGTT-----T** **AGTAAGGTCC**

*S. erinacei* D64072.1 **..........** **..........** **......----** **---.......** **......--..** **..........** **.......-..** **....-----.** **..........**

*S. erinaceieuropaei* KX528090 **..........** **..........** **......----** **---.......** **......--..** **..........** **.......-..** **....-----.** **..........**

*S. erinaceieuropaei* KY552801 **..........** **..........** **......----** **---.......** **......--..** **..........** **.......-..** **....-----.** **..........**

*D. latum* KF218247 **..........** **..........** **......----** **---.......** **......--..** **..........** **.......-..** **....-----.** **..........**

*D. latum* KF218246.1 **..........** **..........** **......----** **---.......** **......--..** **..........** **.......-..** **....-----.** **..........**

*D. latum* DQ925309 **..........** **..........** **......----** **---.......** **......--..** **..........** **.......-..** **....-----.** **..........**

*T. pisiformis* JX317675.1 **G..AGAT..C** **.CG..CAT..** **A.G.CCCATC** **ATG...CGTG** **GTT.TTGTT.** **.....GT.G.** **..TGC.TA..** **TA..ACAAC.** **T..-T...GT**

*T. krabbei* MH843684.1 **G..C...CT.** **...AT.TA..** **TTTA..ATGT** **TGG....T.T** **CTG.TT--TA** **ATAT..T.GT** **T..A.A.T..** **AAG.-----.** **T..GTAA.TT**

100 110 120 130 140 150 160 170 180

....|....| ....|....| ....|....| ....|....| ....|....| ....|....| ....|....| ....|....| ....|....|

Badger *Meles meles*1B_1 **..........** **..........** **...-......** **..........** **-----.....** **..........** **..........** **-.........** **..........**

Badger *Meles meles*1B_2 **..........** **..........** **...-......** **..........** **-----.....** **..........** **..........** **-.........** **..........**

Badger *Meles meles*1B_3 **..........** **..........** **...-......** **..........** **-----.....** **..........** **..........** **-.........** **..........**

**Badger *Meles meles*2B_1**  **..........** **..........** **...-......** **..........** **-----.....** **..........** **..........** **-.........** **..........**

**Badger *Meles meles*2B_2** **..........** **..........** **...-......** **..........** **-----.....** **..........** **..........** **-.........** **..........**

**Badger *Meles meles*2B_3** **..........** **..........** **...-......** **..........** **-----.....** **..........** **..........** **-.........** **..........**

**Badger *Meles meles*2B_4** **..........** **..........** **...-......** **..........** **-----.....** **..........** **..........** **-.........** **..........**

**Badger *Meles meles*2B_5** **..........** **..........** **...-......** **..........** **-----.....** **..........** **..........** **-.........** **..........**

Wild boar *Sus scrofa*1D **..........** **..........** **...-......** **..........** **-----.....** **..........** **..........** **-.........** **..........**

Wild boar *Sus scrofa*2D **..........** **..........** **...-......** **..........** **-----.....** **..........** **..........** **-.........** **..........**

Wild boar *Sus scrofa*3D **..........** **..........** **...-......** **..........** **-----.....** **..........** **..........** **-.........** **..........**

Grass snake *Natrix natrix*41G **..........** **..........** **...-......** **..........** **-----.....** **..........** **..........** **-.........** **..........**

Grass snake *Natrix natrix*53G_1 **..........** **..........** **...-......** **..........** **-----.....** **..........** **..........** **-.........** **..........**

Grass snake *Natrix natrix*53G_2 **..........** **..........** **...-......** **..........** **-----.....** **..........** **..........** **-.........** **..........**

Grass snake *Natrix natrix*53G_3 **..........** **..........** **...-......** **..........** **-----.....** **..........** **..........** **-.........** **..........**

**Otter *Lutra lutra*W1_1** **..........** **..........** **...-......** **..........** **-----.....** **..........** **..........** **-.........** **..........**

**Otter *Lutra lutra*W1_2** **..........** **..........** **...-......** **..........** **-----.....** **..........** **..........** **-.........** **..........**

**Raccoon dog *N.procyonoides*J1_1** **..........** **..........** **...-......** **..........** **-----.....** **..........** **..........** **-.........** **..........**

**Raccoon dog *N.procyonoides*J1_2**  **..........** **..........** **...-......** **..........** **-----.....** **..........** **..........** **-.........** **..........**

**Fox *Vulpes vulpes*Lis 1** **..........** **..........** **...-......** **..........** **-----.....** **..........** **..........** **-.........** **..........**

**Fox *Vulpes vulpes*Lis_2** **..........** **..........** **...-......** **..........** **-----.....** **..........** **..........** **-.........** **..........**

**Am. mink *Neovison vison*1N_1** **..........** **..........** **...-......** **..........** **-----.....** **..........** **..........** **-.........** **..........**

**Am. mink *Neovison vison*73L_1b** **..........** **..........** **...-......** **..........** **-----.....** **..........** **..........** **-.........** **..........**

**Am. mink *Neovison vison*73L_2** **..........** **..........** **...-......** **..........** **-----.....** **..........** **..........** **-.........** **..........**

**Am. mink *Neovison vison*73L_4** **..........** **..........** **...-......** **..........** **-----.....** **..........** **..........** **-.........** **..........**

**Pine marten *Martes martes*51L_1** **..........** **..........** **...-......** **..........** **-----.....** **..........** **..........** **-.........** **..........**

**Pine marten *Martes martes*51L_2** **..........** **..........** **...-......** **..........** **-----.....** **..........** **..........** **-.........** **..........**

**Pine marten *Martes martes*54L_1** **..........** **..........** **...-......** **..........** **-----.....** **..........** **..........** **-.........** **..........**

**Pine marten *Martes martes*54L_2** **..........** **..........** **...-......** **..........** **-----.....** **..........** **..........** **-.........** **..........**

**Pine marten *Martes martes*54L_3** **..........** **..........** **...-......** **..........** **-----.....** **..........** **..........** **-.........** **..........**

**Pine marten *Martes martes*54L_4** **..........** **..........** **...-......** **..........** **-----.....** **..........** **..........** **-.........** **..........**

**Polecat *Mustela putorius*50L_1** **..........** **..........** **...-......** **..........** **-----.....** **..........** **..........** **-.........** **..........**

**Polecat *Mustela putorius*50L_2** **..........** **..........** **...-......** **..........** **-----.....** **..........** **..........** **-.........** **..........**

**Polecat *Mustela putorius*50L_3** **..........** **..........** **...-......** **..........** **-----.....** **..........** **..........** **-.........** **..........**

**Polecat *Mustela putorius*50L_4** **TCGGATTGGC** **GCCATTGCAG** **TGT-CAGCCG** **CAAGGTTGGT** **-----GCTCG** **ACAGGTGCCG** **AGAAGACGAC** **-CAAACTTGA** **TCATTTAGAG**

*S. erinacei* D64072.1 **..........** **..........** **...-......** **..........** **-----.....** **..........** **..........** **-.........** **..........**

*S. erinaceieuropaei* KX528090 **..........** **..........** **...-......** **..........** **-----.....** **..........** **..........** **-.........** **..........**

*S. erinaceieuropaei* KY552801 **..........** **..........** **...-......** **..........** **-----.....** **..........** **..........** **-.........** **.........-**

*D. latum* KF218247 **..........** **..........** **.A.-......** **..........** **-----.....** **..........** **..........** **-.........** **..........**

*D. latum* KF218246.1 **..........** **..........** **.A.-......** **..........** **-----.....** **..........** **..........** **-.........** **..........**

*D. latum* DQ925309 **..........** **..........** **.A.-......** **..........** **-----.....** **..........** **..........** **-.........** **..........**

*T. pisiformis* JX317675.1 **.GATG...AT** **.TTGG..TTT** **G..AAG.AG.** **T....AGCAC** **CAGCCA.ATC** **.GC.ACAA.A** **G.T.TTAAGT** **AAGT.GCAA.** **.G.GC..C..**

*T. krabbei* MH843684.1 **.ATT.G.TAA** **....AGT.TA** **...G.T..TT** **AT..AAGTA.** **TCAT-..GTT** **..TTTAATAA** **..TTTTA.TT** **GT..G.ACT.** **.T..A.TT..**

190 200 210 220

....|....| ....|....| ....|....| ....|....| ..

Badger *Meles meles*1B_1 **..........** **..........** **---...-...** **..........** **..**

Badger *Meles meles*1B_2 **..........** **..........** **---...-...** **..........** **..**

Badger *Meles meles*1B_3 **..........** **..........** **---...-...** **..........** **..**

**Badger *Meles meles*2B_1** **..........** **..........** **---...-...** **..........** **..**

**Badger *Meles meles*2B_2** **..........** **..........** **---...-...** **..........** **..**

**Badger *Meles meles*2B_3** **..........** **..........** **---...-...** **..........** **..**

**Badger *Meles meles*2B_4** **..........** **..........** **---...-...** **..........** **..**

**Badger *Meles meles*2B_5** **..........** **..........** **---...-...** **..........** **..**

Wild boar *Sus scrofa*1D **..........** **..........** **---...-...** **..........** **..**

Wild boar *Sus scrofa*2D **..........** **..........** **---...-...** **..........** **..**

Wild boar *Sus scrofa*3D **..........** **..........** **---...-...** **..........** **..**

Grass snake *Natrix natrix*41G **..........** **..........** **---...-...** **..........** **..**

Grass snake *Natrix natrix*53G_1 **..........** **..........** **---...-...** **..........** **..**

Grass snake *Natrix natrix*53G_2 **..........** **..........** **---...-...** **..........** **..**

Grass snake *Natrix natrix*53G_3 **..........** **..........** **---...-...** **..........** **..**

**Otter *Lutra lutra*W1_1** **..........** **..........** **---...-...** **..........** **..**

**Otter *Lutra lutra*W1_2** **..........** **..........** **---...-...** **..........** **..**

**Raccoon dog *N.procyonoides*J1_1** **..........** **..........** **---...-...** **..........** **..**

**Raccoon dog *N.procyonoides*J1_2** **..........** **..........** **---...-...** **..........** **..**

**Fox *Vulpes vulpes*Lis 1** **..........** **..........** **---...-...** **..........** **..**

**Fox *Vulpes vulpes*Lis_2** **..........** **..........** **---...-...** **..........** **..**

**Am. mink *Neovison vison*1N_1** **..........** **..........** **---...-...** **..........** **..**

**Am. mink *Neovison vison*73L_1b** **..........** **..........** **---...-...** **..........** **..**

**Am. mink *Neovison vison*73L_2** **..........** **..........** **---...-...** **..........** **..**

**Am. mink *Neovison vison*73L_4** **..........** **..........** **---...-...** **..........** **..**

**Pine marten *Martes martes*51L_1** **..........** **..........** **---...-...** **..........** **..**

**Pine marten *Martes martes*51L_2** **..........** **..........** **---...-...** **..........** **..**

**Pine marten *Martes martes*54L_1** **..........** **..........** **---...-...** **..........** **..**

**Pine marten *Martes martes*54L_2** **..........** **..........** **---...-...** **..........** **..**

**Pine marten *Martes martes*54L_3** **..........** **..........** **---...-...** **..........** **..**

**Pine marten *Martes martes*54L_4** **..........** **..........** **---...-...** **..........** **..**

**Polecat *Mustela putorius*50L_1** **..........** **..........** **---...T...** **..........** **.-**

**Polecat *Mustela putorius*50L_2** **..........** **..........** **---...T...** **..........** **.-**

**Polecat *Mustela putorius*50L_3** **..........** **..........** **---...-...** **..........** **..**

**Polecat *Mustela putorius*50L_4** **GAAGTAAAAG** **TCGTAACAAG** **---GTT-CCG** **TAGGTGAACC** **TG**

*S. erinacei* D64072.1 **..........** **..........** **---...-...** **..........** **..**

*S. erinaceieuropaei* KX528090 **..........** **..........** **---...T...** **..........** **..**

*S. erinaceieuropaei* KY552801 **----------** **----------** **----------** **----------** **--**

*D. latum* KF218247 **..........** **..........** **---...T...** **..........** **..**

*D. latum* KF218246.1 **..........** **....G.....** **---...T...** **..........** **..**

*D. latum* DQ925300 **..........** **..........** **---...T...** **..........** **A.**

*T. pisiformis* JX317675.1 **A.G.AG.G..** **.GC...TG..** **AAT...-AAC** **.GT...CGGT** **G.**

*T. krabbei* MH843684.1 **..CT......** **.AA.GTT..A** **TTA...TGTT** **A.T.....AT** **AA**
