# Supplementary material for: Multispecies reservoir of Spirometra erinaceieuropaei (Cestoda: Diphyllobothridae) in carnivore communities in north-eastern Poland
Source: Parasit Vectors. 2020 Nov 10;13:560. doi: 10.1186/s13071-020-04431-5 (PMC7654582; doi:10.1186/s13071-020-04431-5)

**Multispecies reservoir of *Spirometra erinaceieuropaei* (Cestoda: Diphyllobothridae) in carnivore communities in north-eastern Poland**

Eliza Kondzior^1,2*^, Rafał Kowalczyk^1^, Małgorzata Tokarska^1^, Tomasz Borowik^1^, Andrzej Zalewski^1^, Marta Kołodziej-Sobocińska^1^

^1^ Mammal Research Institute, Polish Academy of Sciences, Stoczek 1, 17-230 Białowieża, Poland

^2^ Faculty of Biology, University of Białystok, Ciołkowskiego 1J, 15-245 Białystok, Poland

**Additional file 3: Figure S2.** Maximum likelihood phylogenetic tree of 225 bp sequences of 18S RNA gene fragment based on 38 sequences of *Spirometra* sp. individuals extracted from mammal and reptile species and reference GeneBank achieved sequences: *Spirometra erinacei* (D64072.1), *Taenia krabbei* (MH843684.1), and *Taenia pisiformis* (JX317675.1). The sequences acquired in this study are in bold. *Spirometra* DNA sequences from European badger, wild boar, and grass snake had been described by Kołodziej-Sobocińska et al. [32,40] and by Kondzior et al. [9]. The maximum likelihood method is based on the Tamura-Nei model (1993) applied in MEGA6 software [55]. The tree is drawn to scale, with branch lengths measured in the number of substitutions per site.


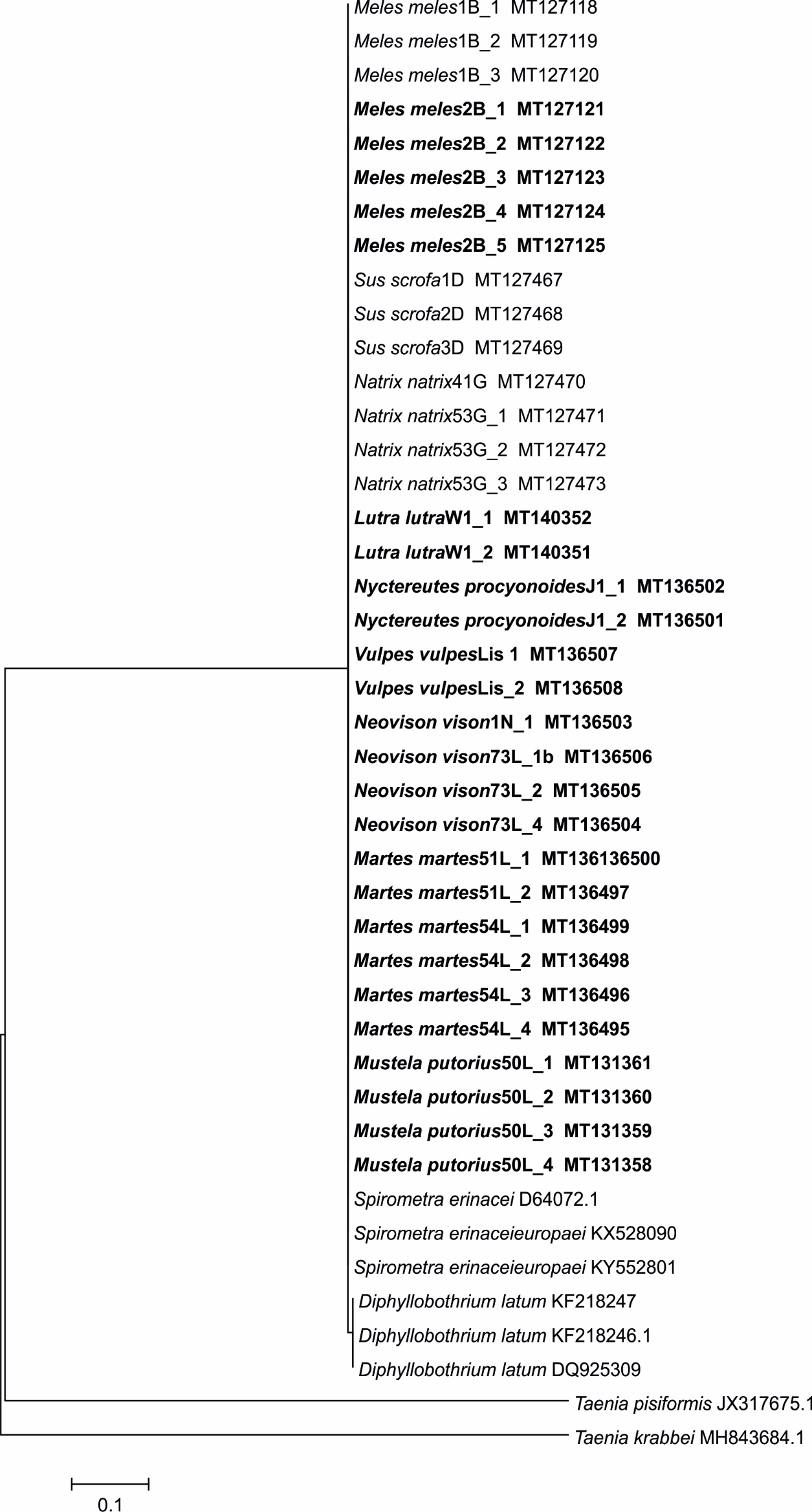

Supplement: Supplementary file 3 — Additional file 3: Figure S2. Maximum likelihood phylogenetic tree of 225 bp sequences of the 18S RNA gene fragment based on 38 sequences of Spirometra sp. individuals extracted from mammal and reptile species and reference sequences retrieved from GenBank: Spirometra erinacei (D64072.1); Taenia krabbei (MH843684.1); and Taenia pisiformis (JX317675.1). The sequences generated in the present study are indicated in bold. Spirometra DNA sequences from the European badger, wild boar, and grass snake had been published by Kołodziej-Sobocińska et al. [32, 40] and Kondzior et al. [9]. The tree is drawn to scale, the scale-bar indicates the number of substitutions per site. [file 13071_2020_4431_MOESM3_ESM.docx]
